# Construction of microbial systems for polyethylene terephthalate degradation

**DOI:** 10.1016/j.synbio.2025.07.013

**Published:** 2025-07-29

**Authors:** Dingkun He, Yichen Gong, Mingzhu Ding, Yingjin Yuan

**Affiliations:** a State Key Laboratory of Synthetic Biology, School of Synthetic Biology and Biomanufacturing, Tianjin University, Tianjin, China; b Frontiers Science Center for Synthetic Biology (Ministry of Education), Frontiers Research Institute for Synthetic Biology, Tianjin University, Tianjin, China

**Keywords:** Polyethylene terephthalate, Microbial degradation systems, Characterization methods, Synthetic biology

## Abstract

Polyethylene terephthalate (PET) is one of the most widely used plastic materials, and its large-scale application has caused severe environmental pollution. Compared to traditional physical and chemical degradation methods, biological degradation is considered the most promising approach. In this context, this review starts with the current research status of PET plastic microbial degradation. Then, it summarizes the construction of strains for heterologous expression of PET-degrading enzymes, the development status of whole-cell catalysts, the innovative ideas of microbial consortia and microorganism-enzyme systems, and the application development of microorganism-functional material systems. In addition, this review includes the use of multiple characterization methods to monitor the degradation effects of PET and changes in strain characteristics, providing theoretical evidence for PET degradation research. Finally, the researches on valorization of PET degradation monomers through synthetic biology are discussed, underscoring the potential of microbial degradation in PET waste upcycling.

## 1 Introduction

Plastics, as a crucial synthetic material in modern society, have brought great convenience to human life. PET is one of the most popular plastic materials used for packaging [1]. The global annual PET production has surged to tens of millions of tons and continues to grow. Given its environmental persistence and low biodegradability, PET accumulates in ecosystems long-term. Furthermore, PET-derived microplastics (1 μm-5 mm) and nanoplastics (<1 μm) present serious threats to both environmental and human health [2].

**Fig. 1:**
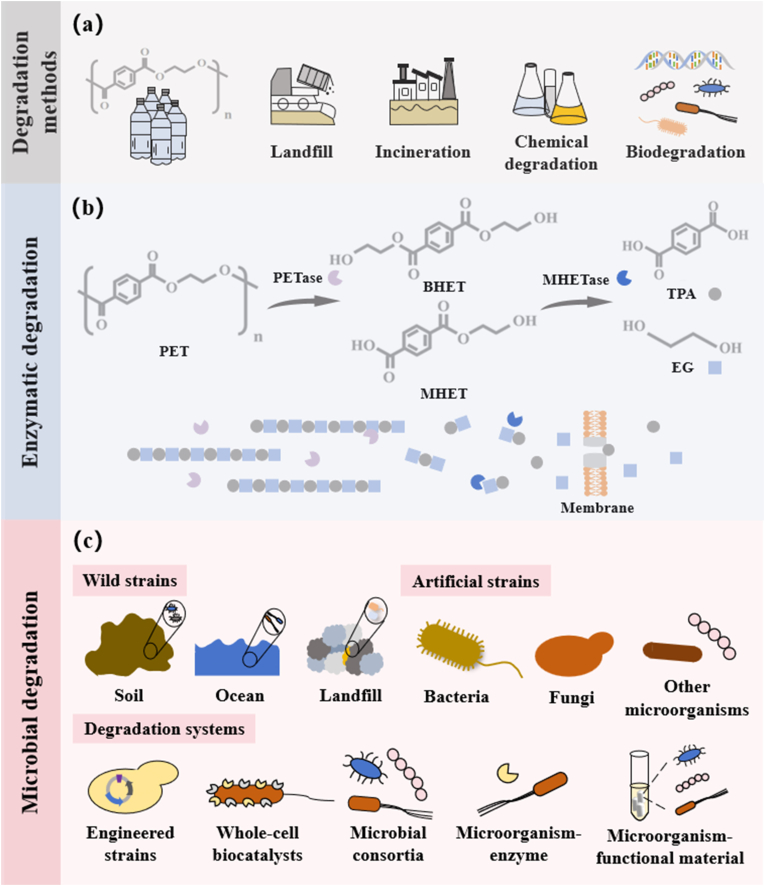
Degradation strategies for PET: emphasis on biological pathways and mechanisms. (a) Different degradation methods of PET. (b) The enzymatic degradation mechanism and process of PET. (c) The microbial degradation systems of PET. TPA: terephthalic acid; EG: ethylene glycol.

The conventional methods for recycling of PET principally involve mechanical, thermal, chemical and other aspects [[3], [4], [5]]. However, simple landfilling can further pollute the environment, especially groundwater [6]. Burning PET will release various pollutants that threaten the respiratory system, such as bisphenol A, dioxins, polychlorinated biphenyls, furans, etc. The cost of catalysts and product separation for chemical degradation is relatively high [7]. Therefore, in recent years, biodegradation has garnered significant interest due to its mild, efficient and low-energy consumption characteristics [8,9], bringing new hopes for dealing with PET waste (Fig. 1a).

Biodegradation methods include enzymatic and microbial degradation [10]. As reported in the literature, as early as 1977, several commercially available lipases and one esterase had been confirmed to possess the ability to hydrolyze multiple polyesters [11]. Müller et al. [12] mark a milestone in PET biodegradation by first reporting the lipase derived from *Thermobifida fusca*. In 2016, Yoshida et al. [13] first isolated *Ideonella sakaiensis* 201-F6 from nature, a bacterium capable of growing on PET as its sole carbon source. The PETase and MHETase produced by it can degrade lcPET films at 30 °C, converting PET to bis (2-hydroxyethyl) terephthalate (BHET) and mono (2-hydroxyethyl) terephthalate (MHET) (Fig. 1b), and ultimately to terephthalic acid and ethylene glycol [14]. As research progresses, numerous enzymes capable of degrading PET have been identified, encompassing keratinases, esterases, hydrolases, and lipases [15]. In addition, direct extraction of total DNA from environmental samples to construct metagenomic databases and screen for enzymes has proven effective for studying unculturable microorganisms. The discovery of leaf and branch compost cutinase (LCC) from metagenomic data serves as a typical example [16].

The catalytic activity of PET hydrolases is crucial for their industrial applications. Researchers are committed to enhancing the activity of PET-degrading enzymes through various bioengineering strategies, such as rational design, irrational design and semi-rational design [10]. After six rounds of mutation and the introduction of disulfide bonds, HotPETase, which possesses exceptional catalytic activity, has a T_m_ increased to 82.5 °C, allowing it to operate below the T_g_ of PET [17]. Based on structural analysis of the enzyme, rational design was employed in the development of LCC-ICCG [18], LCC-RIP [19], LCC-ICCG.I6M [20], LCC-A3 [21] and LCC-YGA [22], which have significantly enhanced the activity and thermostability of PET-degrading enzymes. Notably, machine learning-aided engineering played a pivotal role in the development of engineered PETase variants, DuraPETase and FASTPETase [23].

Microbial degradation, though less efficient than enzymatic degradation, avoids complex enzyme purification and allows reuse in multistep reactions (Fig. 1c). During the 1970s, researchers first observed the colonization of plastics by microorganisms [24]. Subsequently, strains from genera such as *Pseudomonas*, *Rhodococcus*, *Bacillus*, *Penicillium*, and *Aspergillus* have been successively screened and confirmed to effectively degrade PET [3]. Currently, various bacteria, fungi and algae that can interact with PET have been discovered in marine environments, landfills, soil or composting habitats [25,26]. Recent years have seen growing research on efficient PET-degrading microorganisms, with increasing focus on constructing PET-degrading microbial consortia and microorganism-enzyme systems [[27], [28], [29]]. Concurrently, characterization techniques are undergoing breakthrough development from traditional ex situ analysis to in situ dynamic monitoring [30].

Since PET cannot directly enter cells for biodegradation, microbial depolymerization begins with PET-degrading microorganisms attaching to the surface and forming biofilms. These biofilms secrete extracellular enzymes that break down PET into ethylene glycol and terephthalic acid. The potential hazard of ethylene glycol and terephthalic acid persisting in the environment has driven the upcycling of PET [31,32]. Microbial factories achieve the valorization of monomers derived from PET degradation by establishing pathways. But there are many unresolved issues regarding their practical application, such as low expression levels or challenges in scaling up to industrial levels. Therefore, an increasing number of studies are focusing on modifying model chassis organisms through synthetic biology and metabolic engineering to address the multi-gene requirements in the degradation and upcycling of PET [33,34].

Most existing reviews on PET biological degradation focus on the catalytic properties, degradation mechanisms, efficiency optimization of PET hydrolases, and metabolic studies of wild-type strains, with few systematic reviews on engineered strains. This paper mainly focuses on the construction strategies of PET degradation systems from five innovative dimensions, systematically summarizes the multi-scale characterization methods for changes in PET degradation effects and in strain characteristics, and simultaneously emphasizes the application potential of in situ characterization techniques in the dynamic monitoring of PET degradation. Additionally, it reviews research advancements in the valorization of PET monomers using synthetic biology technologies and metabolic engineering approaches as the core, providing systematic professional references for researchers in this field.

## 2 Innovative strategies for enhancing PET microbial degradation

Microbial degradation of PET holds promise for sustainable plastic upcycling but is constrained by multiple bottlenecks: the rigid aromatic structure and high crystallinity of PET impede enzyme-substrate interactions; natural PET-degrading enzymes exhibit low catalytic efficiency and poor stability; accumulation of toxic products (e.g., terephthalic acid) inhibits microbial activity; and single strains struggle to adapt to complex environments (e.g., mixed plastics, environmental fluctuations) [35]. Synthetic biology and metabolic engineering address these challenges via five strategies: constructing heterologous expression strains to enhance enzyme production, developing whole-cell catalysts for cost-effective catalysis, leveraging microbial consortia for synergistic degradation of PET and mixed plastics, integrating enzymatic and microbial systems to improve reaction efficiency, and combining microorganisms with functional materials to optimize substrate accessibility and catalytic performance.

### 2.1 Construct strains by synthetic biology techniques

The imperative for extensive PET degradation has driven the scientific community to explore microbial strains that can highly express PET-degrading enzymes. When constructing strains using synthetic biology, the structural characteristics of PET (rigid aromatic ester backbone, high crystallinity, and hydrophobic surface) serve as the core basis for designing engineering strategies (Fig. 1b). The degradation of PET relies on the specific cleavage of ester bonds by enzymes, which provides clear targets for the application of synthetic biology tools [36].

Host strains engineered via synthetic biology have achieved functional expression of multiple PET-degrading enzymes, and the interaction between host traits and PET significantly affects degradation efficiency. *Escherichia coli* as a classic expression host, has successfully heterologously expressed 9 endogenous PET-degrading enzymes from *Stenotrophomonas pavanii* JWG-G1 [37]. *Bacillus subtilis*, with strong secretion capacity and stress tolerance, is used to express enzymes such as IsPETase, BhrPETase, and LCC; its secretion system enhances the contact efficiency between enzymes and solid PET substrates, making it particularly suitable for degrading high-crystallinity PET [38]. Non-model microorganisms, advantageous in adapting to complex environments, are more suited to practical PET degradation scenarios. *Chlamydomonas reinhardtii* and *S*. *pavanii* engineered to enhance biofilm-forming ability, can increase contact area with PET surfaces through physical adsorption, compensating for enzyme-substrate binding barriers caused by PET hydrophobicity [37,39].

**Fig. 2:**
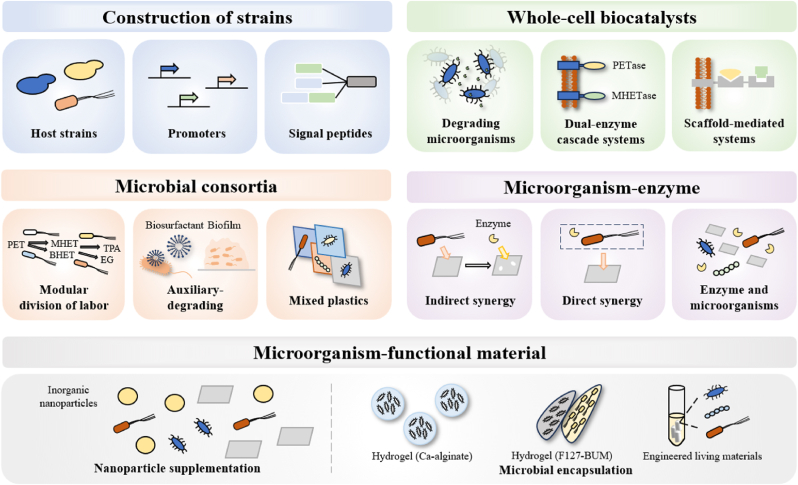
Construction strategies for PET microbial degradation systems.

Host selection and genetic optimization significantly impact the expression and stability of PET-degrading enzymes, necessitating precise alignment with the molecular requirements of PET (Fig. 2). For the requirement of high-temperature PET degradation (to overcome crystallinity), post-translational modifications in different hosts can enhance enzyme thermostability. Glycosylation of LCC in *Pichia pastoris* increases its aggregation temperature by 10 °C, significantly reducing the risk of inactivation at high temperatures [40]. TfCut2 expressed in *B. subtilis* has a more stable spatial structure than the same enzyme expressed in *E. coli*, making it more resistant to local microenvironmental fluctuations during PET degradation [41]. Optimization of promoters and signal peptides directly enhances enzyme targeting to PET (Fig. 2). In *E. coli*, the B1PelB signal peptide increases IsPETase secretion by 62-fold, strengthening the directed transport of enzymes to the PET surface [42]. Co-expression of molecular chaperones (e.g., with IsPETase and LCC) can assist enzyme folding to maintain the conformation of their active centers, ensuring efficient cleavage of PET ester bonds [38,43]. Future research should prioritize the exploration of non-model microorganisms as hosts and the optimization of host cell expression systems.

### 2.2 Construct whole-cell biocatalysts to degrade PET

The expensive and complex purification steps have become an important barrier to the commercial application of PET-degrading enzymes [15]. Whole-cell catalysts eliminate the need for cell lysis or resource-intensive protein purification. Using intact microbial cells as catalytic units, they degrade PET via their intrinsic enzyme systems and metabolic networks, serving as an economical, efficient, and reusable alternative [[44], [45], [46], [47]].

The application of whole-cell biocatalyst in PET degradation demonstrates their diverse potential, offering innovative solutions for plastic pollution in different environments. A biocatalyst from *Comamonas testosteroni* CNB-1 can depolymerize PET microplastics at ambient temperature, achieving 9 % degradation efficiency within 7 days [48]. In response to the unique marine environment, the diatom *Phaeodactylum tricornutum* has been utilized as an effective host for PETase expression, enabling PET degradation in a saline environment at 21 °C, offering a promising strategy for addressing marine pollution [49]. In industrial high-temperature applications, the thermophilic anaerobic bacterium *Clostridium thermocellum* expresses LCC at high levels at 60 °C, highlighting the potential for high-temperature PET degradation [50]. Importantly, whole-cell catalysts not only provide a protective environment for multiple enzymes in metabolic pathways within cells (Fig. 2), but also integrate host metabolic networks to convert degradation products into high-value chemicals [48].

Whole-cell biocatalysts are limited by the inability of secreted enzymes to accumulate on the PET surface [51]. Cell surface display technology, using synthetic biology to anchor enzymes to the outer membrane (Fig. 2), overcomes this by enhancing enzyme-substrate interactions [52]. PETase has been successfully displayed on the surface of *P. pastoris* [53] and *E. coli* [54]. Dual-enzyme cascade catalysis has also been successfully applied such as PETase-MHETase [51] and FastPETase-MHETase [55]. Co-displaying PET-degrading enzymes with certain functional auxiliary proteins on the cell surface has become an optimization direction for surface display, like hydrophobin HFBI on yeast [56]. However, challenges remain in displaying larger proteins on the surface. Scaffold-mediated surface display system can more effectively utilize cell surface space and enhance the synergistic effect between enzymes (Fig. 2). But the stability of its assembly is currently the main reason limiting its large-scale application [57,58].

### 2.3 Construct microbial consortia to degrade PET

Microbial consortia, with their adaptability and metabolic versatility, effectively overcome the limitations of single strains [[59], [60], [61], [62]]. Most microbial consortia are derived from wild microbial communities and are constructed through experimental techniques such as community enrichment, functional community screening, and combinatorial assessment [63]. Compared to wild microbial communities, these artificial systems feature simpler compositions, clearer division of labor, and more efficient degradation pathways [27,64].

The division of labor within microbial consortia (Fig. 2) can be modularized based on PET degradation pathways, with each module assigned to specific strains [65]. Artificial enrichment of mangrove ecosystem has yielded two consortia with PET degradation capabilities demonstrating this modular architecture. *Mangrovimarina plasticivorans* hydrolyzes MHET, while terephthalic acid is converted through protocatechuic acid pathways mediated by *Rhodopseudomonas palustris*. Ethylene glycol is transformed into acetaldehyde via the combined actions of *Pseudoxanthomonas winnipegensis*, *Kaistia cartagenensis*, and *Mycolicibacterium fortuitum* [66]. Furthermore, advances in synthetic biology have enabled the construction of metabolic networks by combining engineered microorganisms with distinct functional modules [27]. For example, a two-species consortium of *B. subtilis* secreting PETase and MHETase achieves higher depolymerization rates than monocultures, while integration of terephthalic acid-metabolizing *Rhodococcus jostii* modules further improves the degradation efficiency [67].

The integration of auxiliary-degrading functional strains into microbial consortia enhances PET degradation through cooperative mechanisms (Fig. 2). For instance, synthetic consortia combining *Lysinibacillus*, *Paenibacillus*, *Gordonia* and *Cupriavidus* spp. exhibit the ability to produce biosurfactants, which enhance substrate bioavailability by emulsifying hydrophobic polymers [68]. Applying the biosurfactant production module to the microbial consortium for PET degradation would be a feasible approach. Additionally, the biofilm formed by fungi can serve as microbial scaffolds, providing attachment sites for other microorganisms [69,70]. However, challenges in degrading plastics with artificial microbial consortia remain inevitable. For instance, diverse biological characteristics within the system lead to instability of bacterial communities, genetic mutations in engineered microbial populations, and difficulties in precisely controlling population composition, among others.

The microbial community, with its complex metabolic network and synergistic effects, exhibits enormous potential for degrading mixed plastics [71]. Researches have shown that certain marine microbial communities can not only decompose PET and PE mixtures by secreting degradation enzymes and forming biofilms [59], but also mineralize poly (butylene adipate-co-terephthalate) based copolyesters into carbon dioxide and biomass through multi enzyme synergy and complex metabolic network [72]. These findings reveal the synergistic mechanism of microbial community degradation of composite plastic materials, providing important theoretical basis for the development of efficient biodegradation technologies (Fig. 2).

### 2.4 Construct microorganism-enzyme systems to co-degrade PET

Enzymatic degradation is widely used with relatively high efficiency, yet ∼10 % of PET is non-biodegradable. While microorganisms can fully degrade gram-scale PET annually, they are rather slow. The microbial-enzyme synergy, however, is a powerful strategy, promising to be a key trend in green biotechnology advancement [28].

Synergistic PET degradation strategies operate through two complementary mechanisms, indirect cooperation and direct cooperation. Indirect approaches utilize microbial pretreatment to modify polymer surfaces (Fig. 2), as demonstrated by *S. pavanii* JWG-G1 that increased subsequent enzymatic efficiency by 91.4 % weight reduction [73]. Direct synergy integrates microbial catalysis with enzymatic action. A notable example is the combination of *Microbacterium oleivorans* (secretes the lipase Lip_19-8_) and *T*. *fusca* cutinase that counteract the inhibition of ethylene glycol terephthalate [74]. Thermophilic systems show particular promise, with *Bacillus thermoamylovorans* JQ3 and the leaf-branch compost cutinase variant (ICCG) complexes achieving complete PET degradation under high substrate loads [75].

Emerging innovations integrate enzymes with microbial consortia (Fig. 2). Research has demonstrated that the synergistic action of enzymes and microbial communities facilitates complete PET mineralization, as enzymatic degradation generates more bioavailable intermediates for microbial utilization [76]. Additionally, enzymes can alter the relative abundance of microbial species. For example, the introduction of magnetic biochar-immobilized PET hydrolase reshapes the functional distribution of soil microbiota and alters soil nitrogen and phosphorus cycles. This highlights the potential of microorganism-enzyme systems to enhance PET degradation efficiency and environmental sustainability [77].

### 2.5 Construct microorganism-functional material systems to enhance PET degradation

Innovative synergistic technologies have shown great potential in improving the degradation efficiency of polymer degradation systems [78]. Microorganism-functional material systems accelerate plastic degradation synergistically via nanoparticle supplementation and microbial encapsulation: inorganic nanoparticles (e.g., barium titanate, iron oxide) enhance the activity of PE-degrading bacterial consortia through surface catalysis or electron transfer mechanisms, addressing bottlenecks of substrate recalcitrance and low efficiency [79]; while smart material-mediated encapsulation protects microorganisms from environmental stresses via physical barriers and stabilizes microbial communities, overcoming issues of microbial loss, poor adaptability, and mixed-culture imbalance (Fig. 2). Microbial immobilization strategies using calcium alginate beads effectively has proven effective [80]. Additionally, using F127-BUM hydrogel or engineered living materials to compartmentalize microorganisms constructs a spatially separated microbial consortium, providing higher stability for complex microbial co-culture systems [81,82].

## 3 Characterization methods for the effect of PET microbial degradation

### 3.1 Characterization of PET

#### 3.1.1 Characterization of surface morphology

**Fig. 3:**
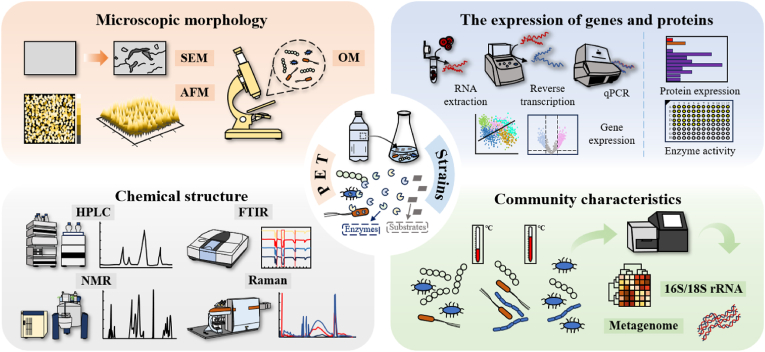
Multi-scale characterization method atlas for PET degradation systems.

Mass loss, while widely utilized as a primary metric for evaluating PET degradation efficiency, fails to comprehensively reflect systemic performance due to its inability to capture microstructural and physicochemical transformations [83,84]. In practical research, surface microscopic morphological observations (Fig. 3) are necessary [85].

Optical microscopy (OM) provides rapid, cost-effective screening but the limited resolution restricts detailed surface analysis [86]. For nanoscale characterization, scanning electron microscopy (SEM) achieves superior resolution (1 nm) and enables multi-angle visualization of degradation-induced microstructural alterations, such as crack propagation and pore formation [17,37]. When coupled with energy-dispersive spectrometer, it can conduct qualitative and quantitative analyses of the elemental composition of samples [87]. Atomic force microscopy (AFM) can employed to analyze microscopic surface morphology changes of plastics under different treatment conditions, particularly roughness and degradation features [23]. Surface roughness serves as a key indicator for assessing PET degradation efficiency [88]. AFM measurements revealed that PET samples subjected to “UV-pretreated and bio-treated” conditions exhibited the highest surface roughness, with a maximum peak-to-valley distance of approximately 0.25 μm, superior to other conditions [88].

#### 3.1.2 Characterization of chemical structure

##### 3.1.2.1 Characterization of PET monomers

For the detection and characterization of PET monomers, high-performance liquid chromatography (HPLC) and nuclear magnetic resonance (NMR) spectroscopy can be selected (Fig. 3). As a highly sensitive and selective analytical technique, HPLC not only detects degradation intermediates such as ethylene glycol, terephthalic acid, MHET and BHET [19,20,23,89], but also analyzes their subsequent metabolic conversion products, like glycolic acid, protocatechuic acid, pyrogallol and so on [32,33,36,[90], [91], [92]]. This capability makes HPLC a critical tool for elucidating the complete pathways of PET biodegradation.

NMR spectroscopy probes PET molecular bonds through nuclear spin interactions in strong magnetic fields [93]. It includes ^1^H and ^13^C NMR [94]. ^1^H NMR analysis shows that the characteristic peak of terephthalic acid is at 7.66 ppm, the doublet peaks at 7.77 ppm and 7.85 ppm indicate MHET, and the peak at 7.97 ppm belongs to BHET [61,95]. This technique permits real-time tracking of degradation product concentrations within detection limits, complementing HPLC analyses [96]. For example, in a recent study on the complete decomposition of PET using a crude PET hydrolase produced, ^1^H NMR was employed to confirm the recovery of terephthalic acid with a purity exceeding 98 % from hydrolyzed PET products, thereby validating the efficiency of the enzymatic degradation process and the purity of the recovered terephthalic acid [89]. On the other hand, NMR technology contributes to the study of PET degradation mechanisms (Fig. 3). Researchers have revealed the molecular mechanism of PET degradation by thermophilic polyester hydrolase TfCut2 through Solution NMR and molecular dynamics simulations, revealing the generation pathways of degradation intermediates and final products [97]. In addition, NMR has been used for quantitative analysis of PET microplastic pollution in environmental samples, providing a powerful tool for assessing the potential impact of PET on ecosystems [98].

##### 3.1.2.2 Characterization of chemical bonds

The characterization of chemical bonds typically employs fourier transform infrared (FTIR) spectroscopy and Raman spectroscopy. FTIR spectroscopy identifies structural changes in PET by measuring its absorption of infrared light [99]. Specific chemical bonds in PET, such as C–O–C (1100-1250 cm^−1^) and C=O (1710-1740 cm^−1^), absorb distinct infrared wavelengths, producing unique “fingerprint peaks” [100]. The strength of key absorption peaks can indicate the degradation of PET plastics by microorganisms [101]. Variations in peak positions or the appearance of new peaks such as O–H at 3764 cm^−1^ can indicate structural modifications caused by treatments like UV exposure, degradation or additives [100]. In addition, combining multivariate methods, such as principal component analysis, enhance structural evaluation by identifying patterns across multiple peaks (Fig. 3). For instance, ratios of absorption bands at 898-973 cm^−1^ and 1340-1370 cm^−1^ effectively differentiate biodegradable from non-biodegradable PET based on crystallinity differences [101].

As a non-destructive chemical analysis technique (Fig. 3), Raman spectroscopy features high resolution, sensitivity to non-polar bonds, and the ability to directly measure samples in complex environments [30,90,102,103]. The characteristic peaks of PET in Raman spectra are primarily found at 1617 cm^−1^ (bending vibration of aromatic rings) and 1730 cm^−1^ (stretching vibration of the carbonyl C=O group) [87]. These peaks not only provide detailed chemical information but also enable high-precision detection and classification in complex environments. They have been successfully used to identify PET in microplastics from various water samples, including those from the North Sea, the Thames River and the Elbe River [102]. When combined with multivariate analysis methods such as principal component analysis and linear discriminant analysis, Raman spectroscopy can distinguish PET from other plastics like polypropylene and polyvinyl chloride [103].

##### 3.1.2.3 In situ characterization of PET

Surpassing the technical limitations of traditional “ex situ characterization”, the cutting-edge in situ characterization techniques demonstrate core value in enabling real-time dynamic monitoring, non-destructive analysis, and multidimensional data acquisition of degradation processes.

At the molecular level, Raman spectroscopy and ATR-FTIR track ester bond cleavage and functional group dynamics to clarify degradation pathways [104]. Structurally, digital holography and confocal Raman microscopy visualize biofilm-induced surface erosion and selective degradation of crystalline-amorphous regions [30]. In complex environments, surface-enhanced Raman spectroscopy and machine learning enable nanoscale fragment detection [105]. With machine learning and multimodal sensors, in situ techniques will overcome detection limits and facilitate PET biodegradation translation to engineering.

In summary, the selection of analytical methods for PET and its degradation products depends on research objectives and sample characteristics. HPLC is preferred for high-precision separation and quantitative analysis of complex mixtures due to its high sensitivity and suitability for low-concentration detection [89]. NMR is ideal for rapid structural identification of pure components, resolving unknown compounds, or studying enzyme-substrate interactions [89,97]. Raman spectroscopy excels in analyzing aqueous samples or systems requiring simultaneous detection of polar/non-polar bonds [102,103]. FTIR is more efficient for rapid identification of polar functional groups, like C=O and O–H, as well as analysis of dried films/powder samples [30]. Driven by the collaborative integration of technologies and the expansion of application scenarios, in situ characterization techniques have become the core bridge connecting fundamental research and applied research on plastic degradation [105].

### 3.2 Characterization of PET-degrading strains

#### 3.2.1 Characterization at gene and protein expressions

Genetic-level studies on PET degradation reveal the differences in microbial gene expression under various conditions and their regulatory mechanisms. By using methods such as real-time fluorescence quantitative polymerase chain reaction and reverse transcription polymerase chain reaction to compare the gene expression differences of microorganisms under different conditions (Fig. 3), genes that are specifically expressed or whose expression levels change significantly during the PET degradation process can be screened out [90,106,107]. These genes are very likely to encode enzymes or regulatory proteins directly related to PET degradation. The degradation and upcycling of PET by microorganisms is a complex biological process that involves the coordinated expression and regulation of multiple genes, including the core genes *Gox0313* and *aldA* for the conversion of ethylene glycol to glycolic acid [91], as well as key genes *TphA*, *TphB*, and *TphC* for the conversion of terephthalic acid to protocatechuic acid [108], among others (Fig. 3). Through gene expression measurement techniques such as transcriptome sequencing, the interaction relationships among genes can be comprehensively understood, including the regulation of target genes by transcription factors and the activation of signal transduction pathways during the degradation process [109]. This not only helps to construct a complete gene regulation model for PET degradation and upcycling, but also provides guidance for the modification of engineered strains [90,91].

At the protein level, the characterization and analysis of PET-degrading enzymes are crucial for understanding their functions and mechanisms. The protein expression level, as a key indicator, can directly reflect the abundance of enzymes. Enzyme activity serves as a direct indicator for evaluating microbial degradation capacity (Fig. 3), particularly in the study of PET-degrading enzymes, where the hydrolysis of specific substrates is commonly used to reflect enzyme activity [110]. P-nitrophenyl butyrate is one of the widely used model substrates [37,111,97,112]. Upon enzymatic hydrolysis, p-nitrophenyl butyrate releases p-nitrophenol, which exhibits a yellow color under alkaline conditions and can be quantified by measuring absorbance at 405–420 nm. In addition, turbidimetric assays [113], fluorescence spectroscopy [114], UV absorbance [115] and HPLC [116] can also be used to determine enzyme activity [91]. These diverse approaches provide robust tools for characterizing and optimizing PET-degrading enzymes.

#### 3.2.2 Characterization at the community level

Molecular techniques have significantly advanced the study of plastic biodegradation by enabling detailed investigations of microbial communities (Fig. 3). The application of 16S/18S rRNA gene sequencing has become a cornerstone for analyzing microbial community structure and succession dynamics [[117], [118], [119], [120], [121]]. For example, distinct microbial community shifts have been identified on marine plastic surfaces and in landfill soils under varying environmental conditions [121]. Through the identification of dominant microbial strains via 16S rRNA sequencing, researchers can subsequently integrate functional gene analysis to predict their biodegradation potential. Understanding microbial environmental adaptability and community stability is particularly critical for practical applications such as PET biodegradation. Operational taxonomic units, constructed based on genetic sequence similarity, streamline microbial community analysis while revealing relationships between environmental factors and microbial interactions, including symbiosis, competition, and cooperation among species [117,119].

Metagenomic approaches, particularly whole metagenome sequencing (Fig. 3), provide comprehensive insights into microbial metabolic capabilities, accelerating the discovery of novel plastic-degrading enzymes and microorganisms [122]. Recent studies have successfully combined metagenomics with computational predictions to identify numerous uncharacterized genes and potential PET-degrading enzymes in plastic-polluted soil samples. Using machine learning models, researchers have ultimately identified 21 novel PET-degrading enzymes [123]. These advancements underscore the power of molecular techniques in deciphering complex plastic biodegradation mechanisms and developing effective bioremediation solutions.

## 4 Upcycling of PET plastics

**Fig. 4:**
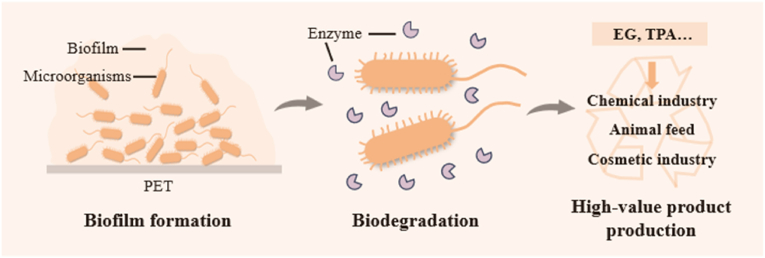
From PET waste to value: a microbial-driven circular degradation pathway.

Addressing pollution issues within the framework of the plastic circular economy is a crucial branch for achieving the sustainable development goals. The highly reactive hydroxyl groups of ethylene glycol, along with the rigid benzene ring and active carboxyl groups of terephthalic acid, endow them with unique reactivity and functional potential, providing a material basis for valorization (Fig. 4). Furthermore, leaks of high concentrations of ethylene glycol and terephthalic acid can lead to persistent environmental risks, which constitutes the core driving force for their upcycling [92].

Multiple wild microorganisms have been identified for their ability to metabolize ethylene glycol. Glycolic acid, as a primary product of ethylene glycol biovalorization, has been extensively studied. *E. coli* [111,91,124], *Yarrowia lipolytica* [125], *Gluconobacter oxydans* [[126], [127], [128]] and other microorganisms have been engineered to produce glycolic acid from ethylene glycol. Our previous study found that engineered *E. coli* MG1655 can achieve a glycolic acid titer of 10.9 g/L, with a yield of 1.13 g/g [91]. Ethylene glycol can also be utilized by *Pseudomonas putida* KT2440 to produce polyhydroxyalkanoates under nitrogen limited conditions [129]. Besides, some studies have also utilized ethylene glycol to produce biodegradable plastics such as medium-chain-length polyhydroxyalkanoates [130] as well as important amino acids like tyrosine [131] and threonine [132]. The yield remains to be further improved.

Terephthalic acid catabolic pathways have been described in *Comamonas testosteroni* [133], *Rhodococcus* sp. [67], *Pseudomonas umsongensis* [134], *Acinetobacter baylyi* [135] and *P*. *putida* [129,136]. Unlike ethylene glycol, terephthalic acid requires specific transport proteins to enter microbial cells [137]. Once inside, terephthalic acid undergoes hydroxylation and dehydrogenation to form protocatechuic acid, a key intermediate in the catabolism of aromatic compounds [33]. From protocatechuic acid, several high-value products can be derived, including gallic acid, pyrogallol, catechol, muconic acid and vanillic acid [127,138,139], as well as downstream products like polyhydroxybutyrate [36], vanillin [140] and lycopene [141]. Notably, engineered strains of *E. coli* and *P. putida* have been successfully developed to convert terephthalic acid into β-ketoadipic acid [142,143], which can be further utilized to produce biodegradable plastics such as polyhydroxyalkanoates [136]. In addition, a dual strain system of engineered *E. coli* has enabled efficient conversion of terephthalic acid to 2-pyrone-4,6-dicarboxylic acid [144].

Most PET valorization research remains confined to the laboratory scale, with a gap from industrial application. A key bottleneck is the low efficiency of PET biodegradation, which severely hinders subsequent monomer valorization. To improve degradation efficiency, a sequential strategy combining chemical degradation and biotransformation can be adopted. Meanwhile, leveraging community synergy to convert ethylene glycol and terephthalic acid into high-value products represents a core direction for future development [145]. Furthermore, exploring innovative product portfolios enabling co-transformation of ethylene glycol and terephthalic acid is an important approach to expand PET monomer utilization. By breaking through traditional monomer utilization models and developing novel metabolic pathways, ethylene glycol and terephthalic acid can be co-transformed within the same system into new products (e.g., lycopene [146], tyrosine [131]) with unique properties and market potential. This not only enhances the economic value of PET monomers but also provides more diverse and competitive product options for industrial applications, thereby facilitating the transition of PET monomer valorization from laboratory research to industrial-scale application.

## 5 Summary and outlook

This paper systematically reviews the cutting-edge advancements in the field of microbial degradation of PET, deeply analyzes the innovative design logic of enzymatic and microbial degradation systems, and innovatively proposes a multi-dimensional strategy evaluation framework with in situ characterization technology as the frontier technology. In addition to the extensive research on the upcycling of PET into high-value chemicals, energy production from PET waste has emerged as a novel approach, reflecting broader concerns for sustainable resource management and environmental protection.

As research progresses, the field of PET microbial degradation is poised for further innovation. The exploration of marine and extremophilic microorganisms is emerging as a promising frontier for discovering novel PET-degrading resources. The selection and engineering of host cells for efficient expression of PET-degrading enzymes will remain crucial. Future research could focus on optimizing host cell expression systems, employing synthetic biology to construct more efficient microbial factories, or delving into non-model microorganisms to develop advanced degradation systems. The construction of microorganism-enzyme systems or microbial consortia holds potential for enhanced degradation efficiency. Integrating machine learning to model and design synthetic consortia could enable precise control over microbial proportions, thereby enhancing system stability and efficiency. The upcycling of PET degradation monomers is essential for achieving resource sustainability and environmental protection. The development of more efficient enzymes and industrial-scale applications will drive the integration of biological recycling into the circular economy. Additionally, bioelectrochemical systems have been increasingly applied in the degradation of polyethylene, polylactic acid and polyvinyl chloride, holding promise for providing a novel approach for PET circularity. As synthetic biology and metabolic engineering continue to advance, the future of PET biodegradation is expected to become more efficient and cost-effective, significantly aiding sustainable plastic waste management.

## CRediT authorship contribution statement

**Dingkun He:** Writing – review & editing, Writing – original draft, Visualization, Methodology, Investigation, Conceptualization. **Yichen Gong:** Visualization, Methodology, Investigation, Conceptualization. **Mingzhu Ding:** Writing – review & editing, Supervision, Funding acquisition, Conceptualization. **Yingjin Yuan:** Supervision.

## Declaration of competing interest

The authors declare that they have no known competing financial interests or personal relationships that could have appeared to influence the work reported in this paper.

## Data Availability

No data was used for the research described in the article.
